# Magnetic Induction Heating Enables On-Demand Drug Release via Diels–Alder Polymeric Nanocarriers

**DOI:** 10.1021/acs.biomac.5c00321

**Published:** 2025-10-23

**Authors:** Nanami Fujisawa, Mitsuhiro Ebara, James J. Lai

**Affiliations:** 1 Research Center for Macromolecules and Biomaterials, 52747 National Institute for Materials Science, Tsukuba 305-0044, Japan; 2 Graduate School of Pure and Applied Sciences, University of Tsukuba, Tsukuba 305-8577, Japan; 3 Department of Materials Science and Engineering, 34878 National Taiwan University of Science and Technology, Taipei 10607, Taiwan; 4 Department of Bioengineering, University of Washington, Seattle, Washington 98195, United States

## Abstract

As a stimulus-responsive drug release system, we developed Diels–Alder (DA) induction-activated magnetic nanoparticles (DiMaN). Functionalization of polymer-coated magnetic nanoparticles (mNPs) with drugs via thermoreversible DA coupling enabled release through retro-Diels–Alder (rDA) cleavage upon heating. The (pDMAm-*co*-pFMA)-*b*-pAAc polymer supported mNP formation and drug binding. DA coupling with maleimide-functionalized drugs was verified by ^1^H NMR, showing distinct exo (3.23 ppm) and endo (3.48 ppm) signals after 72 h at 37 °C. Approximately 70% release was observed within 15 min at 80 °C, while the complex maintained stability at 40 °C. The superparamagnetic mNPs generated localized heating under an alternating current magnetic field (192 kHz, 480 A), raising the solution temperature by 6 °C within 5 min. The biotin-maleimide complex demonstrated higher release by rDA from furan-containing mNPs (approximately 150 μM) compared to the control group (approximately 103 μM). These results highlight DiMaN as a promising platform for magnetic-controlled, on-demand drug release.

## Graphical abstract

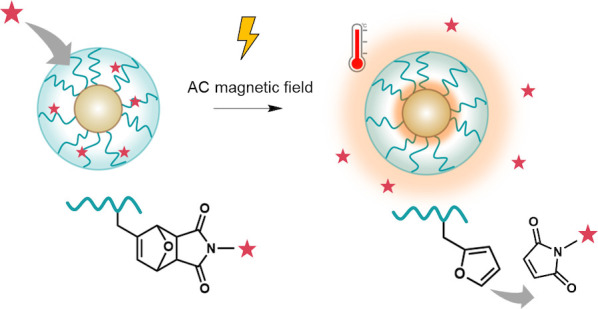

## 1 Introduction

Infusion pumps are widely used for the controlled subcutaneous or intravenous administration of drugs, particularly in insulin and opioid treatments.^1^ These devices enable precise and programmable drug delivery, allowing for home-based therapy and long-term medication management.^2,3^ The U.S. Food and Drug Administration (FDA) has issued alerts regarding the risks of over- or underdosing, drug leakage, and system malfunctions associated with infusion pumps.^4^ To ensure patient safety, accurate programming, precise dosage control, and proper user training have been emphasized as critical factors in the effective use of these devices.^5,6^ Another major limitation of infusion pumps is their reliance on continuous drug exposure, which may not always align with the body’s natural biological rhythms or optimal drug efficacy windows.^7^ In some cases, treatments benefit from precise spatiotemporal control over drug activation, such as aligning drug release with the circadian cycle or responding to specific physiological conditions.^8^

Given these challenges, there is a need to develop safer, more reliable, and externally controllable drug delivery systems that minimize user error while maintaining precise therapeutic control.^9^ One promising approach is the use of externally triggered, on-demand drug release platforms, which allow for localized drug activation in response to external stimuli such as temperature, light, or magnetic fields.

Ideally, an on-demand drug delivery system would not require constant external intervention but instead utilize an implantable material capable of externally triggered, precisely controlled drug release.^10^ Spatial control of drug therapy requires active selection of the treatment site, which can be achieved with locally implanted systems.^11^ Additionally, an on-demand drug release mechanism would allow precise temporal control over drug administration at any given time.^5^ Several temperature-responsive drug release systems have been investigated, including those based on temperature-sensitive polymers (e.g., poly­(*N*-isopropylacrylamide), pNIPAAm),^12^ hydrogen bonding (e.g., DNA),^13^ and disulfide bonding.^14^ However, physical phase transitions alone may not provide sufficient accuracy, as drugs may still be released at lower temperatures.^15^ Drug release systems that respond to pH changes have also been developed, often designed to take advantage of the tumor microenvironment.^16^ While such approaches can be effective, they are not ideal for conditions like epilepsy, diabetes, or cancers requiring synchronization with circadian rhythms.^17,18^ Among potential control mechanisms, temperature stands out as a promising trigger because it remains stable at approximately 37 °C in the human body. Consequently, if temperature changes can be externally induced, drug release could be controlled with high precision. For example, gold nanoparticles and near-infrared (NIR) light have been used as heat sources to trigger drug release;^13^ however, these approaches face limitations when targeting deep tissues due to the poor penetration of external stimuli.

In this study, we hypothesize that DiMaN (Diels–Alder induction-activated magnetic nanoparticles), magnetic nanoparticles (mNPs) decorated with polymers containing furan groups, can serve as a temperature-responsive on-demand drug release system when exposed to an AC magnetic field (Scheme 1). DiMaN incorporates drug molecules with maleimide derivatives by conjugating to the polymer’s furan groups via the Diels–Alder (DA) reaction, allowing for stable attachment.^19^ Upon exposure to an AC magnetic field, the magnetic core of the nanoparticles would generate localized heat,^20^ triggering retro-Diels–Alder (rDA) cleavage to release the conjugated drug molecules.^21^ To test this hypothesis, we synthesized a diblock copolymer, poly­(dimethylacrylamide-*co*-furfuryl methacrylate)-*block*-poly­(acrylic acid), (pDMAm-*co*-pFMA)-*b*-pAAc, which serves as a template for mNP synthesis. The pAAc block coordinates iron cations via carboxylate complexation, facilitating the formation of iron oxide particle cores,^22^ while the pDMAm-*co*-pFMA block maintains the resulting particles’ colloidal stability. Beyond nanoparticle characterization, their inductive heating behavior was confirmed under an AC magnetic field. The model drug molecules with a maleimide derivative were conjugated to the particle by reacting with the furan group of the pDMAm-*co*-pFMA block via DA reaction. Then, the conjugated drug molecules were released by cleaving the thermoreversible covalent bond via the heat-induced rDA reaction using the iron oxide mNPs as a heat source. DiMaN can potentially provide a novel, externally controlled drug delivery system, combining magnetothermal activation and temperature-responsive polymer chemistry for on-demand control of drug release and highly selective and precise therapeutic applications.

**Scheme 1:**
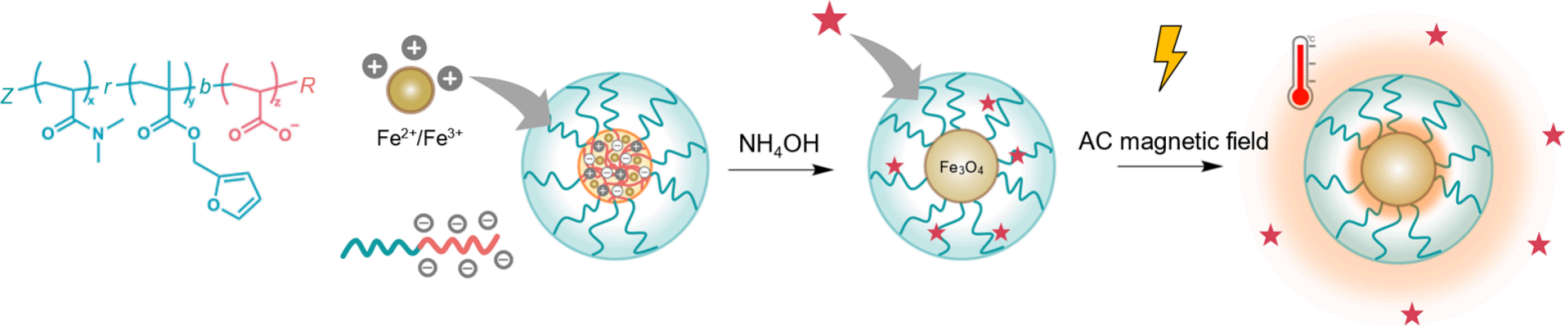
Schematic Diagram to Illustrate the Formation of Polymer-Templated mNPs^a^

a MNPs decorated with (pDMAm-*co*-pFMA) can introduce drug molecules by the DA reaction. When an AC magnetic field is applied to the DiMaN, the drug molecules are released by exotherm of the mNPs, triggering the rDA reaction.

## 2 Materials and Methods

### 2.1 Materials

Azobis­(isobutyronitrile) (AIBN), deuterium oxide (99.8%, for nuclear magnetic resonance (NMR)), lithium chloride (LiCl), and dimethylformamide (DMF) were purchased from FUJIFILM Wako Pure Chemical Corporation (Osaka, Japan). Acrylic acid (AAc), *N,N*-dimethylacrylamide (DMAm), NHS-PEG_2_-maleimide (NHS-PEG_2_-MAL, >98.0%), and 4-cyano-4-[[(dodecyl thio)­carbonothioyl]­thio]­pentanoic acid (CTA) were purchased from Tokyo Chemical Industry Co., Ltd. (Tokyo, Japan). Ultrapure distilled Milli-Q water (Merck, Darmstadt, Germany) was used in this study. Furfuryl methacrylate (FMA), iron­(II) chloride tetrahydrate, iron­(III) chloride hexahydrate, and ammonia solution (28% in water) and biotin-PEG_3_-MAL were purchased from Sigma-Aldrich (MO, USA). The monomethyl ether hydroquinone inhibitors—that is, FMA, AAc, and DMF—were removed by passing them through an alumina oxide column before use.

### 2.2 Block Copolymer Synthesis

For the homopolymer of pDMAm_150_ (without the furan group), 4.00 g (40.351 mmol) of DMAm was added; for the copolymer with the furan group of p­(DMAm_145_-*co*-FMA_5_), 3.781 g (38.146 mmol) of DMAm and 0.2186 g (1.315 mmol) of FMA were added in each 20 mL sample bin. Moreover, 0.1086 g (0.269 mmol) of CTA reagent (the degree of polymerization was 150), 0.0044 g (0.027 mmol) of AIBN (10 wt % versus CTA), and 6.0 mL (30 wt %) of DMF were added in each bin and vortexed well. Each sample was sealed with a rubber cup (14/20 joints, Precision Seal rubber septa, Sigma–Aldrich) and a parafilm. Each monomer solution was purged with N_2_ gas for 20 min. The samples were then polymerized at 70 °C for 4 h; the polymerization was stopped by opening the rubber cup. For polymer purification, the reaction solution was added with enough space in each 3.5k molecular weight cutoff (MWCO) dialysis membrane and dialyzed against 2.0 L of methanol, after which the dialysis solvent was exchanged using 2.0 L of distilled water, the distilled water being exchanged three times. After polymer purification by dialysis, the polymer solution was added to each 120 mL sample bin, prefrozen using liquid N_2_, and then lyophilized (FDL-2000, Tokyo Rikakikai Co., Ltd., Tokyo, Japan) for 3 days to obtain a yellowish-white powder. The polymers obtained in this study were then used as macro-CTAs (mCTA) in the block copolymers. Next, ^1^H NMR spectroscopy at 400 MHz (JEOL Ltd., Tokyo, Japan) was used to confirm the polymer structures by dissolving the polymer in D_2_O at a concentration of 10.0 mg mL^–1^. The molecular weight was determined using gel permeation chromatography (GPC; Nexera 40, Shimadzu Corporation, Kyoto, Japan). The mobile phase was 10 wt % LiCl in DMF at 40 °C, and the elution peaks were detected using an RI detector (Shodex RI-501, Resonac Corporation, Tokyo, Japan). Finally, the Mn, Mw, and polydispersity index (PDI) were calculated using poly­(methyl methacrylate) (PMMA) standards.

### 2.3 Synthesis of Block Copolymer p­(DMAm-*co*-FMA)-*b*-pAAc

For polymerization with acrylic acid (AAc), pDMAm_150_-*b*-pAAc_20_, and p­(DMAm_145_-*co*-FMA_5_)-*co*-pAAc_20_, 2.00 g (0.118 mmol) of each mCTA (pDMAm_150_ and p­(DMAm_145_-*co*-FMA_5_)) was added to the 20 mL samples. A total of 0.17 g (2.360 mmol) of AAc (degree of polymerization = 20), 1.93 mg (0.012 mmol) of AIBN (10 wt % versus mCTA), and 17.36 mL of DMF (10 wt % versus the total reactant mass) were added to each bin and vortexed thoroughly. Each sample was sealed using a rubber cup and parafilm. Each monomer solution was purged with N_2_ gas for 20 min. The samples were then polymerized at 70 °C for 4 h; the polymerization was stopped by opening the rubber cup. To purify the polymers, sufficient space was added to the reaction solution in each 3.5 kDa MWCO dialysis membrane and dialyzed against 2.0 L of methanol, after which the dialysis solvent was exchanged using 2.0 L of distilled water, the distilled water being exchanged three times. After dialysis, the purified polymer solution was added to each 120 mL sample bin, prefrozen using liquid N_2_, and then lyophilized for 3 days to obtain a yellowish-white powder.

### 2.4 Synthesis of Magnetic Nanoparticles

For the synthesis of mNPs using the polymer as a template, synthesized block copolymers 1.25, 2.5, 5, 10, and 20 times the amount of iron­(II) chloride tetrahydrate (FeCl_2_·4H_2_O):iron­(III) chloride hexahydrate (FeCl_3_·6H_2_O) (these being in a 1:2 ratio) were added to the block copolymer carboxylic acid. Specifically, FeCl_2_·4H_2_O and FeCl_3_·6H_2_O were dissolved in Milli-Q water in advance at concentrations of 0.1856 and 0.3712 M, respectively. The block copolymers pDMAm_150_-*co*-pAAc_20_ and p­(DMAm_145_-*co*-FMA_5_)-*b*-pAAc_20_ were also dissolved in Milli-Q water at concentrations of 50.0 mg mL^–1^ each. The aqueous iron chloride solutions, aqueous polymer solutions, and Milli-Q water used for the mNP synthesis were purged using N_2_ gas. Then, 50.0 μL of the polymer solution was added to each 1.5 mL Eppendorf tube and mixed well. An iron chloride solution was added to each volume, as shown in the table (Figure S1) and incubated for 30 min. After incubation, 10 μL of the ammonia solution (28% in water) was added and mixed well before being incubated for 30 min again. The color of the solution changed from transparent to light brown. After 30 min at 25 °C, the solution was filtered with a 0.22 μm poly­(vinylidene difluoride) (PVDF) syringe filter. The mNPs in the filtrate were purified via size exclusion chromatography (SEC) performed with SEC beads (Sepharose CL-4B, Cytiva, Tokyo, Japan) by gravity chromatography with Econo-Pac chromatography columns (Bio-Rad Laboratories, Inc., CA, US). Each 0.5 mL of eluted mNP fraction was collected using a 1.5 mL Eppendorf tube and measured each fraction component at 310 (CTA) and 500 (mNPs) nm by a NanoDrop One^c^ (Thermo Fisher Scientific, Inc., MA, US). Similarly, fourier-transform infrared spectroscopy (FT-IR; IRAffinity-1s, Shimadzu Corporation, Kyoto, Japan) was used to evaluate the composition of polymers and mNPs. Dry polymer powders and dry mNPswere evaluated by attenuated total reflection FT-IR (ATR-FT-IR) method. To estimate the amount of polymer contained in mNPs, mg of eachmNPs (Fe:COOH ratio of 1.25:1, 2.5:1, 5:1, 10:1 and 20:1) sample wa scaled, then change in mass was measured by differential thermal analysis-thermogravimetry (TG-DTA; TG-DTA6200, Seiko Instruments Inc., Chiba, Japan) when the temperature was increased from 25 °C to 550 °C with the increase rate at 10 °C min^-1^. The weight reduction rate was calculated with the pre-measurement weight set as 100%. Hydrodynamic diameter measurements were performed using an ELSZ-2000 instrument (Otsuka Electronics Co., Ltd., Osaka, Japan). A high-power semiconductor laser was used as the incident beam. After the concentration of mNPs (Fe:COOH ratio of 20:1) was adjusted to a level in the measurable range with Milli-Q water, at each layer number, filtered by a 0.45 μm pore size, 13 mm-diameter polytetrafluoroethylene syringe filters (Membrane Solutions, LLC, WA, USA) then added the solution in disposable cuvettes. These samples were used for the particle size measurements at 25 °C. The zeta potential was measured using a standard cell unit (Otsuka Electronics Co., Ltd., Osaka, Japan). Additionally, TEM images of the particles were taken to obtain their appearance. For sample preparation, a sufficiently diluted particle dispersion solution was prepared to obtain an image with well-dispersed particles. Two microliters of the particle dispersion solution were dispensed onto the carbon support film of the TEM grid (HRC-C10 STEM Cu100P, Okenshoji Co., Ltd., Tokyo, Japan) and the water was dried. To prevent particle agglomeration due to rapid water evaporation, the grid was left overnight in a refrigerator with a damp Kimwipe to allow the water to evaporate slowly, yielding a sample suitable for TEM imaging. TEM observation was performed by Talos F200X G2 (Thermo Fisher Scientific Inc., MA, US). TEM images were acquired at an accelerating voltage of 80 kV. For elemental mapping, scanning transmission electron microscopy (STEM) images were acquired with energy dispersive X-ray spectroscopy (EDS) by Super-X.

### 2.5 Heat Generation Behavior

The heating profiles of the freeze-dried mNPs were investigated by applying an AC magnetic field. Polymer-coated mNPs were collected by lyophilization. The sample was placed in the middle of a copper coil and exposed to an AC magnetic field at 192 kHz and 480 A using a HOSHOT2 instrument (Alonics Co., Ltd., Tokyo, Japan). The heating profiles were obtained by capturing photographs using a forward-looking IR camera (CPA-E6, Teledyne FLIR LLC., OR, USA).

### 2.6 Magnetization Curve

The *M*–*H* curve under a DC magnetic field of the mNP in a 0.544 mg/mL water suspension was measured using the physical property measurement system (PPMS) (Quantum Design Inc., CA, US) at room temperature. The mNP suspension was added to a glass tube and placed in the machine.

### 2.7 Diels–Alder Polymer Reaction

The model drug—that is, NHS-PEG_2_-MAL (161.0 mg)—was dissolved in 11.5 mL of D_2_O. The p­(DMAm_145_-*co*-FMA_5_) (100.0 mg) was then placed in the sample bin. The NHS-PEG_2_-MAL solution (1.0 mL) was added to the polymer-containing sample bin and allowed to react under stirring. The reacted sample solution (0.7 mL) was collected at various time points and analyzed using ^1^H NMR spectroscopy. The progress of the DA reaction was plotted as a function of reaction time with respect to the newly formed proton-derived peaks corresponding to the *K*_endo_ (3.48 ppm) and *K*_exo_ (3.24 ppm) products of the DA reaction, which appeared after the reaction.

### 2.8 Retro-Diels–Alder Reaction from the Polymer

For the rDA reaction, the DA reaction solution (0.7 mL) was added to an NMR tube. The ^1^H NMR spectroscopy measurements were performed at different temperatures (40, 60, and 80 °C) at each time point (every 10 min for a total of 30 min). The progress of the rDA reaction was plotted as the percentage of NHS-PEG_2_-MAL remaining in the polymer from the *K*_endo_ (3.48 ppm) and *K*_exo_ (3.24 ppm) products, which were used as references for the DA reaction, for each reaction temperature and time. The plot begins with the introduction ratio of NHS-PEG_2_-MAL to the polymer, which was approximately 60%.

### 2.9 Biotin Conjugates and Releases upon the AC Magnetic Field on Magnetic Nanoparticles

The polymers pDMAm-*b*-pAAc and p­(DMAm-*co*-FMA)-*b*-pAAc (2.5 mg) and the MNPs coated with pDMAm-*b*-pAAc and p­(DMAm-*co*-FMA)-*b*-pAAc (polymer amount to be 2.5 mg) were predissolved in Milli-Q water, and 4.16 mg (6.96 μM) of biotin-PEG_3_-MAL was dissolved in each solution and then incubated for 4 days at 37 °C. To remove excess biotin-PEG_3_-MAL, Milli-Q water was added and concentrated using a 30 kDa Amicon ultracentrifugal filter (3,500 rpm, 20 min). This washing process was performed in triplicate. The purified mNP solution was placed in a 1.5 mL Eppendorf tube under an AC magnetic field (192 kHz, 480 A) by using a HOSHOT2 instrument for 30 min to allow the release of biotin-PEG_3_-MAL. The released biotin-PEG_3_-MAL was collected using an Amicon ultracentrifugal filter (3,500 rpm, 20 min). Next, 20 μL of the biotin-PEG_3_-MAL released sample was added to the well of a 96-well plate, and 180 μL of the HABA/avidin assay mixture (AnaSpec, Inc., CA, US) was added to the same well. The sample was mixed well by shaking it on a plate shaker at 100–200 rpm for 5 min, and the absorbance was read at 500 nm using a plate reader (Infinite 200 PRO, Tecan Group Ltd., Männedorf, Switzerland). The microplate data could be calculated as follows:

$$\Delta A_{500}=A_{500,\mathrm{negative}\;\mathrm{control}}-A_{500,\mathrm{released}\;\mathrm{biotin}\;\mathrm{sample}\;\mathrm{or}\;\mathrm{positive}\;\mathrm{control}}$$ 1

$$\mathrm{biotin}\;\mathrm{concentration}\left(M\right)=\left[\frac{\Delta A_{500\;\mathrm{nm}}}{\left(34,500\times 0.5\right)}\right]\times \mathrm{dilution}\;\mathrm{factor}$$ 2

$$\left(\varepsilon_{\mathrm{HABA}/\mathrm{avidin}}=34,500\;M^{-1},\;\mathrm{light}\;\mathrm{path}=0.5\;\mathrm{cm}\right)$$ 3

## 3 Results and Discussion

### 3.1 Polymer Synthesis

To template the mNP synthesis and facilitate drug incorporation via the DA reaction, this work developed a diblock copolymer, p­(DMAm-*co*-FMA)-*b*-pAAc. The pAAc block coordinates iron cations via carboxylate complexation, facilitating the formation of iron oxide particle cores.^22^ To improve the particles’ colloidal stability, furfuryl methacrylate (furan monomer) was copolymerized with dimethylacrylamide,^23^ p­(DMAm-*co*-FMA). Additionally, the p­(DMAm-*co*-FMA) block also provides furan groups to drug molecule conjugation via the DA reaction. The polymer synthesis is illustrated in Scheme 2. The p­(DMAm-*co*-FMA) was first prepared via reversible addition–fragmentation chain-transfer (RAFT) polymerization of FMA and DMAm in the presence of the chain-transfer agent (CTA) and a radical initiator. In addition to a homo-pDMAm (control), polymers with the DMAm/FMA ratios as 150:0, 145:5, and 140:10 were synthesized. ^1^H NMR spectroscopy was used to confirm the successful polymerization (Figure S1). For p­(DMAm_145_-*co*-FMA_5_), a number-average molecular weight (Mn) of 18,600 Da was determined by GPC (DMF with 10 wt % LiCl) that corresponds to ∼174 monomeric units with a polydispersity index (PDI) around 1.4 (Table S1). The resultant p­(DMAm-*co*-FMA) block was utilized as the macro CTA (mCTA) for the extension with AAc. The GPC and ^1^H NMR results were not able to confirm the chain extension because of the polyelectrolyte nature of pAA; its charged carboxyl groups strongly interact with the stationary phase of the column.^24^ The molecular weight of (pDMAm_145_-*co*-FMA_5_)-*b*-pAAc_7_ was also analyzed using GPC (Figure S2) with DMF containing 10 wt % LiCl as the mobile phase and PMMA standards for calibration. The chromatogram showed two peaks: the peak at 21 min corresponded to Mn ≈ 46 kDa with PDI = 1.1, while the peak at 25 min corresponded to Mn ≈ 6.5 kDa with PDI = 1.3. We suspect that the first peak represents the block copolymer, whereas the second corresponds to the macro CTA, suggesting inefficient chain extension potentially due to partial macro CTA inactivation. However, the theoretical Mn for the block copolymer is 18 kDa, which is significantly lower than the observed 46 kDa. Therefore, an accurate determination of the polymer molecular weight by GPC was not possible in this case. In ^1^H NMR spectroscopy, the proton of the carboxylic acid (−COOH) cannot be quantified because it exchanges with the deuterated solvent to form −COOD and the peak may be lost.^25^ Therefore, titration with a sodium hydroxide aqueous solution was utilized to analyze the incorporated carboxylates (pAAc) quantitatively (Figure S3). The average carboxyl groups per polymer chain were estimated to be seven units.

**Scheme 2:**
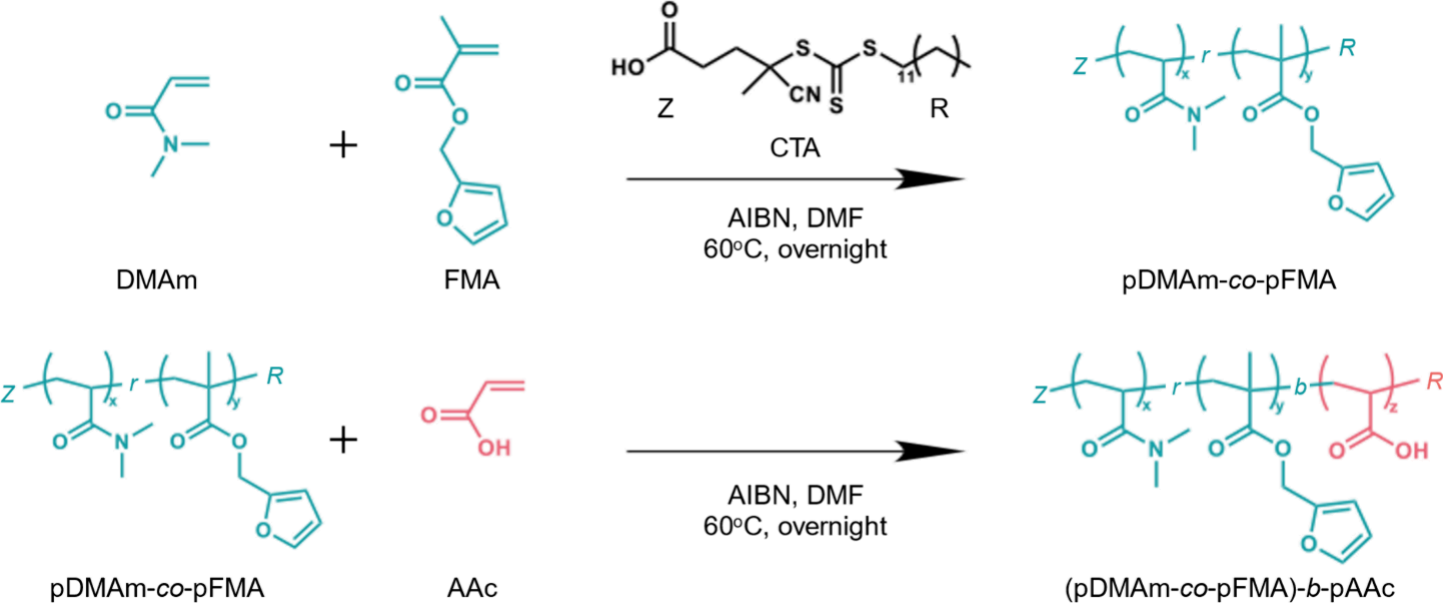
Synthesis Scheme for Polymer Template Particle Synthesis: Copolymerization of Furfuryl Methacrylate (FMA) Containing the Reaction Site of the Diels–Alder Reaction and *N,N*-Dimethylacrylamide (DMAm), a Water-Soluble Monomer, in RAFT Polymerization, Followed by the Introduction of Acrylic Acid as a p­(DMAm-*co*-FMA) Macro CTA^a^

a The block copolymer, p­(DMAm-*co*-FMA)-*b*-pAAc, was synthesized by introducing acrylic acid as a macro CTA.

### 3.2 Synthesis of Magnetic Nanoparticles

Particle synthesis followed the methodology described in our previous study by modifying the polymer design.^22,26,30^ The block copolymer was utilized in the in situ coprecipitation of iron oxide mNPs (Scheme 1). The particles were synthesized by keeping the polymer concentration in the solution constant at 10 mg/mL (5.5 μM acrylic acid) and varying the Fe/COOH ratio from 1.25:1 to 20:1 (6.9–111.3 μM) (Table S2). After the addition of NH_4_OH for inducing iron oxide formation, all reactions resulted in stable colloids except the reactions with a 20:1 Fe/COOH ratio, which resulted in precipitates immediately. The colloidal stable mNPs were purified via size exclusion chromatography (SEC) for further characterizations, including Fourier transform infrared spectroscopy (FT-IR), thermogravimetric-differential thermal analysis (TG-DTA), dynamic light scattering (DLS), and scanning transmission electron microscopy (STEM) with energy-dispersive X-ray spectroscopy (EDS) mapping.

FTIR spectroscopy was performed to confirm the chemical composition of the synthesized polymer and the mNPs. The FTIR spectrum of the polymer exhibited characteristic absorption bands corresponding to its functional groups. A broad peak observed around 3,200–3,600 cm^–1^ was attributed to the O–H stretching vibration, indicative of carboxyl functional groups. In Figure 1a, the presence of strong absorption bands at 1,615 and 1,730 cm^–1^ confirmed the amide stretching (O=C–N) of pDMAm and carbonyl (C=O) stretching of pAAc. The presence of peaks at 1,055 and 1,500 cm^–1^ are assigned to C–O–C and C=C stretching bands in the furan ring.^27^ The mNP spectrum was very similar to the polymer spectrum; however, the absorption band of carbonyl stretching was significantly reduced because the carboxyl groups are embedded in the particle core.

**Figure 1:**
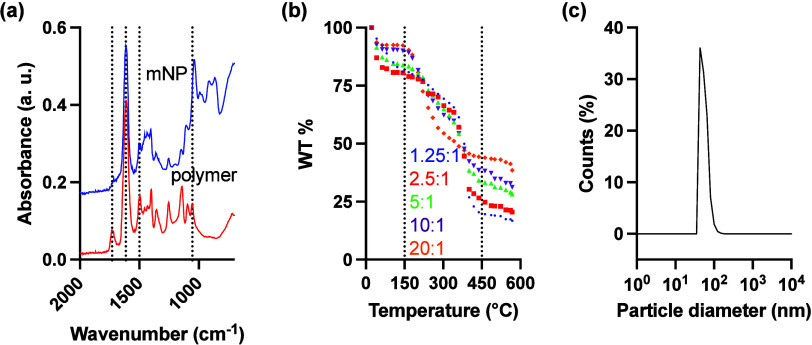
(a) ATR-FT-IR of p­(DMAm-*co*-FMA)-*b*-pAAc (red) and polymer-decorated mNPs (blue), (b) TG-DTA of polymer-decorated mNPs in various Fe:COOH ratios, and (c) hydrodynamic radius of the synthesized mNPs in the ratio of 20:1.

The thermal analysis (Figure 1b), TG-DTA, was conducted to evaluate the thermal stability and composition of the polymer-coated mNPs. The thermogram exhibited a few distinct weight loss regions corresponding to different thermal events. The initial weight loss of approximately 5–20% observed below 150 °C was attributed to the removal of adsorbed moisture from the particle surface. This indicates the presence of physically bound water within the polymer coating. A significant weight loss of approximately 50% occurred between 200 and 500 °C, corresponding to the thermal decomposition of the polymer shell. The major degradation step suggests the breakdown of the polymer backbone, including the cleavage of organic functional groups. Beyond 500 °C, the weight stabilized, indicating that the remaining 15–40% of the sample was composed of thermally stable iron oxide, confirming the presence of the mNP core. These results demonstrate the successful coating of the mNPs with a polymer layer and provide insight into their composition.

DLS was performed to determine the hydrodynamic size distribution of the mNPs. The measurements revealed an average hydrodynamic radius of 55 nm (95% CI: 43–81 nm), Figure 1c. The particles also demonstrated good stability. They were stored at 4 °C for ≥4 months and remained colloidal stable prior to the conjugation and release experiments without observable degradation. In addition, our previous work showed that nanoparticles synthesized using polymeric templates remain stable for more than 2 months while maintaining their particle size, transition temperature, and thermoresponsive magnetic separation behavior.^25^

Transmission electron microscopy (TEM) images of the synthesized mNPs revealed a core–shell structure (Figure 2a (inset)), with darker contrast areas corresponding to the iron oxide core and a lighter contrast layer representing the polymer coating.^33^ To further analyze the size distribution of the particle iron oxide cores, TEM images were used to construct a histogram. The analysis determined an average core size of 7.7 nm (95% CI: 7.0–8.4 nm), Figure 2b. Selected-area electron diffraction analysis (Figure 2c) was performed via TEM to examine the crystalline structure of the synthesized iron oxide nanoparticles. The diffraction pattern exhibited distinct ring-like diffraction features, indicative of a polycrystalline nature.^28^ The observed diffraction rings corresponded to the characteristic lattice planes of magnetite (Fe_3_O_4_) or maghemite (γ-Fe_2_O_3_), with prominent reflections indexed to the (311) and (400) lattice planes.^29^

**Figure 2:**
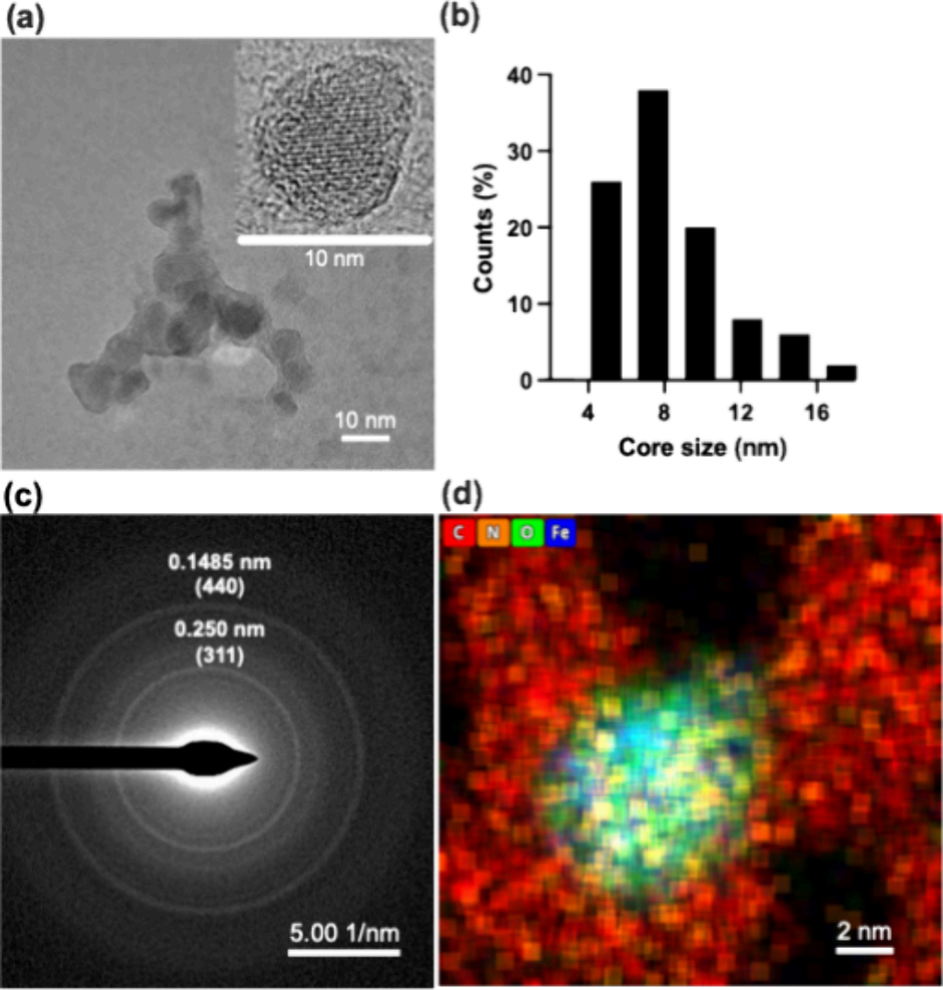
(a) TEM image of the synthesized mNPs, (b) core size histogram of the synthesized mNPs measured via the TEM image, (c) electron diffraction, and (d) EDS mapping image by STEM (c). Blue dots indicate Fe, green dots indicate oxygen, orange dots indicate nitrogen, and red dots indicate carbon.

EDS analysis was performed to determine the elemental composition of the synthesized nanoparticles, focusing on C, N, O, and Fe (Figure 2d). The EDS mapping from STEM revealed a strong overlap of Fe and O, confirming the presence of the iron oxide core. Additionally, N signal was observed, indicating the presence of the polymer coating. The colocalization of Fe, O, and N supports the successful encapsulation of the mNPs with the polymer, further validating the core–shell structure.

Several synthesis methods have been developed for mNPs, including sol–gel,^30^ coprecipitation,^31^ thermal decomposition,^9,32^ microemulsion,^33^ and microwave-assisted techniques.^34^ In this study, we employed a coprecipitation method^2,35^ in aqueous conditions at room temperature. The pAAc block served as a templating agent,^22^ enabling iron cation coordination via carboxylate complexation, followed by oxidation to form Fe_3_O_4_ nanoparticles. To achieve effective inductive heating under an AC magnetic field, Fe_3_O_4_ was preferred over γ-Fe_2_O_3_ because of some reasons.^36^ The synthesis involved FeCl_2_·4H_2_O and FeCl_3_·6H_2_O as precursors, following the reaction:

$${\mathrm{Fe}}^{2+}+2{\mathrm{Fe}}^{3+}+8{\mathrm{OH}}^{-}\to {\mathrm{Fe}}_{3}O_{4}+4H_{2}O$$ 4

Iida et al. demonstrated that Fe_3_O_4_ particle size can be controlled by adjusting the Fe^2+^/Fe^3+^ ratio during coprecipitation.^37^ The iron oxide core size of 7–9 nm observed in this study aligns with previously reported values. The ability to control particle size can potentially be utilized for tuning magnetothermal properties, which will be discussed in the section on heat generation behavior.

The core–shell structure of the synthesized polymer-coated mNPs was confirmed through multiple characterization techniques, including FT-IR, TGA, DLS, TEM, and EDS. The polymer not only facilitated iron oxide nanoparticle formation but also provided colloidal stability and a functional surface for drug conjugation via the DA reaction.

### 3.3 Magnetization Curve and Heat Generation Behavior

The superparamagnetic properties of the synthesized nanoparticles were characterized using vibrating sample magnetometry (VSM) at room temperature over a ±1 T field range (Figure 3a). The saturation magnetization (Ms) at an applied field of 1 T was measured to be 0.15 emu/g, and the magnetization (*H*–*M*) curve exhibited negligible hysteresis, confirming the superparamagnetic nature of the nanoparticles,^38^ which is essential for effective magnetic field-responsive heating and reversible dispersion in solutions.^39^

**Figure 3:**
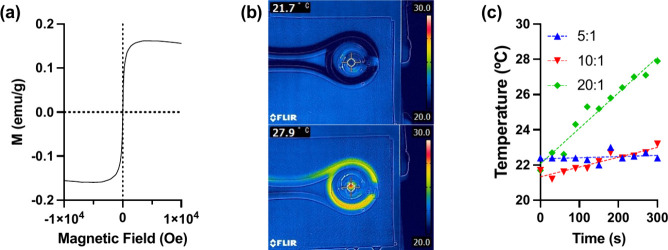
(a) *M*–*H* curve of mNPs in a 0.544 mg/mL water suspension at 298 K. Measured using the physical property measurement system; (b) IR images of particle solution at 21.7 and 27.9 °C with an applied AC magnetic field; (c) heat generation behavior of the synthesized mNPs.

To evaluate the magnetic induction heating capability, nanoparticle solutions (10.0 mg/mL) were subjected to an alternating current (AC) magnetic field at 192 kHz and 480 A. The solution temperature was monitored in real time using an infrared (IR) imaging system (Figure 3b). As shown in Figure 3c, particles synthesized with Fe:COOH ratios of 5:1, 10:1, and 20:1 increased the solution temperature by 0, 1.5, and 6.2 °C, respectively, after 300 s of AC magnetic field application. This trend clearly demonstrates that higher Fe:COOH ratios result in greater heating efficiency, supporting the conclusion that magnetic heating performance is directly correlated with iron content.

Magnetic heating efficiency was directly correlated with the Fe:COOH ratio, as higher iron content resulted in greater heat generation, likely due to an increased magnetic moment density. A previous study demonstrated that the Ms (emu/g) of iron oxide nanoparticles increases with higher iron content.^40^ Consistent with this, we anticipate that nanoparticles synthesized with higher Fe:COOH ratios possess higher Ms values. Heating under an AC magnetic field is directly related to magnetic losses associated with the alignment and relaxation of magnetic moments; therefore, nanoparticles with higher Ms dissipate more heat. This explains the observed increase in heating efficiency with higher iron content.

The heat generation characteristics induced by an alternating magnetic field in the magnetic nanoparticles synthesized in this study are thought to involve both Néel relaxation and Brownian relaxation for the following reasons. The observed heating behavior follows Néel relaxation mechanisms, as expected for nanoparticles of this size (7.7 nm core diameter).^20,40^ In this process, the magnetic moment within the crystal structure rotates in response to the external field, generating heat without significant physical movement of the nanoparticles. In contrast, Brownian relaxation, where heat is generated through physical rotation of particles due to fluid friction, is typically dominant in larger nanoparticles.^3,41^ The size of mNPs plays a critical role in determining their magnetic properties and heating behavior. Bulk Fe_3_O_4_ exhibits ferromagnetic properties, but when reduced to a nanoscale diameter below 15 nm, particles transition to superparamagnetic behavior,^7,38^ which exhibits zero coercivity allowing the magnetic moments to rapidly align and relax in response to an applied AC magnetic field. This property is particularly advantageous for biomedical applications, as it ensures no residual magnetization once the external field is removed, thereby preventing nanoparticle aggregation *in vivo*. However, since Brownian motion was also observed in DLS for this particle, it was suggested that Brownian relaxation, not just Néel relaxation, may be involved in the heat generation of magnetic nanoparticles.

The results demonstrate that magnetic induction heating efficiency is correlated with iron oxide content, where higher Fe/COOH ratios result in greater heat generation. It is important to consider that temperature measurements in this study were obtained using an IR camera, which records the bulk solution temperature rather than the localized temperature at the particle surface. Since heat dissipation occurs rapidly in aqueous environments, nanoparticles with a Fe/COOH ratio of 5:1, showing no significant heating effect in bulk measurements, may still experience significant localized heating at the particle surface. The ability to generate localized heating under an AC magnetic field presents a promising approach for remotely triggering drug release at targeted sites. Additionally, the ability to control iron content in polymer-templated nanoparticle synthesis provides a major advantage for tuning magnetic heating behavior. By modulating nanoparticle composition, heat generation can be optimized for intended applications, while minimizing off-target heating effects.

### 3.4 Conjugation via the DA Reaction and Release via the rDA Reaction

Other than templating the mNP synthesis, the block copolymer was designed to facilitate drug molecule conjugation via the DA reaction by incorporating furan functional groups via the p­(DMAm-*co*-FMA) block.^4,42^ The initial evaluation utilized *N*-hydroxysuccinimide-PEG_2_-maleimide (NHS-PEG_2_-MAL) as a model molecule because the maleimide group can react with the polymer’s furan moieties to form a covalent linkage (Figure 4a). ^1^H NMR spectroscopy was used to monitor the formation of the exo (*K*_exo_) and endo (*K*_endo_) DA adducts, which represent two possible stereoisomeric products of the DA reaction between furan and maleimide.^5,43^ The characteristic signals at 3.23 (*K*_exo_) and 3.48 (*K*_endo_) ppm clearly indicate the formation of the cycloadduct (Figure 4b). These peaks correspond to protons associated with the new carbon–carbon bonds formed during the DA cyclization of furan and maleimide. The appearance of two distinct chemical shifts reflects the presence of both *K*_endo_ and Kexo stereoisomers. While the overlap limits visual clarity, the observed shifts are consistent with previously reported DA conjugation chemistry and support successful conjugation.^43^

**Figure 4:**
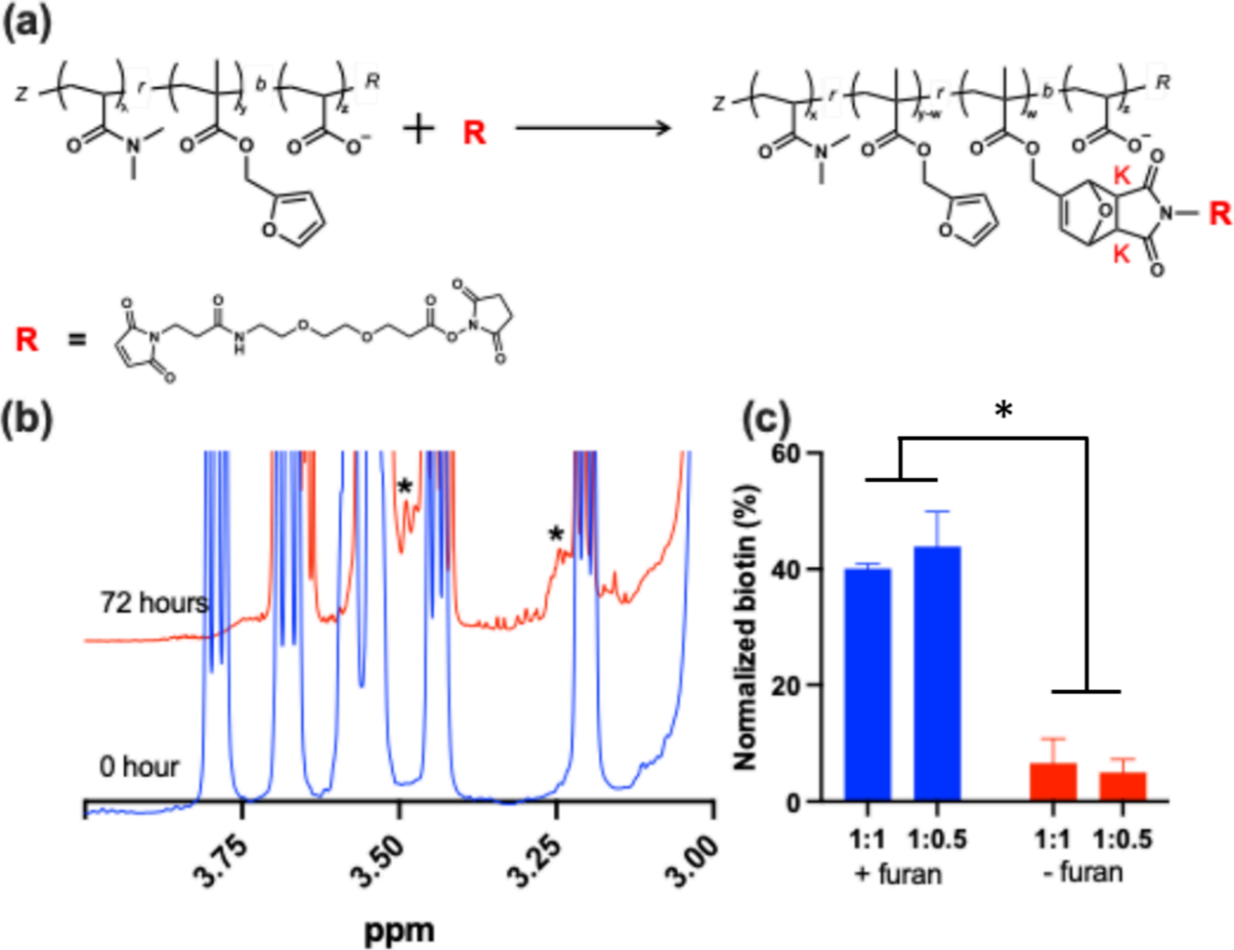
(a) Scheme of Diels–Alder reaction between the polymer and NHS-PEG_2_-MAL, (b) spectra of reaction progress of Diels–Alder reaction by ^1^H NMR (400 MHz), and (c) HABA assay result of biotin-PEG_3_-MAL and polymer conjugation. The furan-containing polymers (blue) resulted in ∼40% conjugation, and the polymers without furan (red) led to <10% nonspecific binding (*p < 0.005).

To demonstrate the Diels–Alder conjugation and retro-Diels–Alder release, maleimide-PEG_2_-biotin was utilized as the model drug molecule. Specifically, polymers containing furan groups were incubated with the biotin solution at two biotin:furan ratios, 1:1 and 1:0.5. After conjugation, free (unconjugated) biotin was removed by membrane filtration (Amicon, 10 kDa MWCO), and the filtrate biotin concentration was measured to estimate the amount of biotin conjugated to the polymer. Compared to the starting biotin solution, a substantial reduction in filtrate biotin confirmed DA conjugation, with furan-containing polymers conjugating ∼40% of biotin, while polymers lacking furan groups showed <10% nonspecific binding under identical conditions (Figure 4c). These results confirm the successful conjugation of NHS-PEG_2_-MAL to the polymer via the DA reaction.

To assess whether heat-induced rDA cleavage could effectively trigger drug release (Figure 5a), NHS-PEG2-MAL-functionalized polymers were incubated at 40, 60, and 80 °C, with ^1^H NMR spectra collected every 5 min to monitor bond dissociation kinetics (Figure 5b). Specifically, the integrations of chemical shifts at 3.23 ppm (Kexo) and 3.48 ppm (*K*_endo_) were used to quantify the extent of NHS-PEG_2_-MAL release over time. The rDA reaction progression was plotted as the percentage of NHS-PEG_2_-MAL remaining in the polymer based on the initial Kexo and *K*_endo_ reference signals. At 80 °C, the release was highly efficient, with approximately 70% of NHS-PEG_2_-MAL released within 15 min. At 60 °C, a moderate release, ∼30%, was observed over 30 min, indicating a temperature-dependent release profile. At 40 °C (near body temperature), no significant bond dissociation was detected, suggesting that the conjugated drug remained stable under physiological conditions.

**Figure 5:**
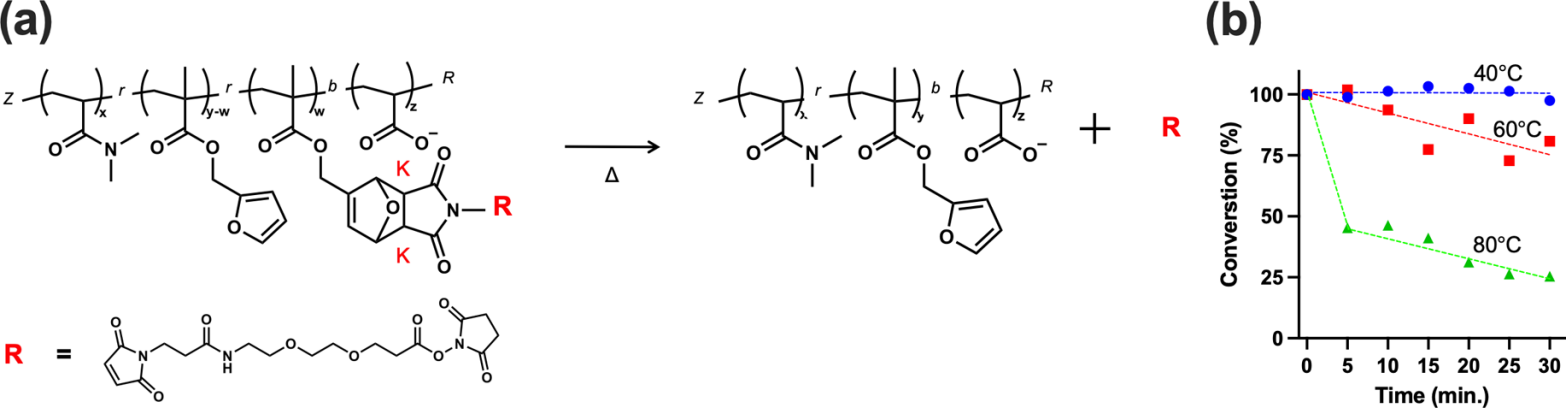
(a) Scheme of retro-Diels–Alder reaction; (b) conversion rate of retro-Diels–Alder reaction at each temperature, calculated from ^1^H NMR measurement.

The successful conjugation of NHS-PEG_2_-MAL to the polymer via the DA reaction and its subsequent temperature-responsive release via the rDA reaction demonstrate the feasibility. The stability of the drug–polymer conjugate at physiological temperatures suggests that unintended premature release is unlikely, which is critical for maintaining therapeutic efficacy and minimizing undesirable systemic exposure. The ability to trigger drug release only when needed offers a distinct advantage over conventional sustained-release systems, which may lack the ability to respond dynamically to changing treatment needs.

While the conjugation of a model maleimide-functionalized drug (biotin-PEG_3_-MAL) to the polymer and its subsequent thermally triggered release have been successfully demonstrated, the next step is to integrate magnetic induction heating with drug release studies. By combining magnetic field-driven heat generation with drug conjugation and release, we aim to establish the feasibility of remotely triggered, localized drug delivery. Biotin-PEG_3_-MAL was conjugated to both the polymers and the magnetic nanoparticles through the furan functional groups via the DA reaction. Biotin release was quantified using the HABA assay (Figure 6). Polymers with and without furan groups served as controls to confirm release via the rDA reaction. Upon heating at 90 °C for 30 min, the average released biotin was nearly 0 μM for polymers without furan and ∼80 μM for polymers containing furan. Under the same heating conditions, particle conjugates released ∼103 μM (without furan) and ∼157 μM (with furan) biotin. When an AC magnetic field (192 kHz/480 A/min) was applied, the particle conjugates released ∼103 μM (without furan) and ∼150 μM (with furan) biotin. Thus, both direct heating and magnetic induction heating triggered significantly higher biotin-PEG_3_-MAL release from furan-containing polymers and particles (by ∼50–80 μM), confirming rDA release. Notably, higher levels of biotin detected in the furan-free particles suggest some degree of nonspecific binding. Nonetheless, the consistent increase in release for furan-containing systems under both direct heating and AC magnetic field exposure demonstrates that induction heating effectively triggered the rDA reaction and subsequent drug release.

**Figure 6:**
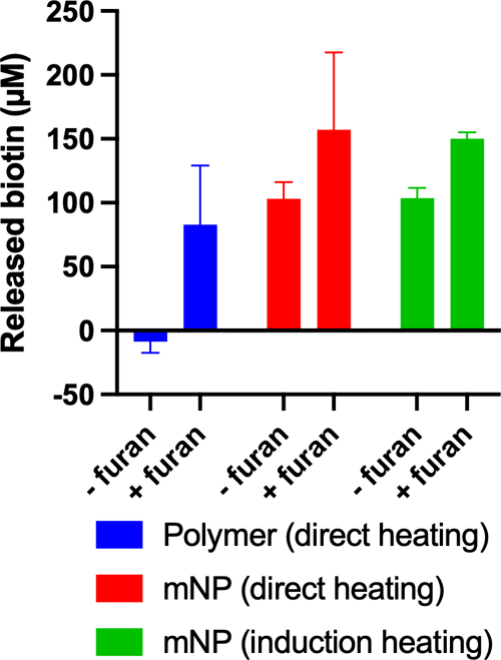
Biotin release profile for polymers with direct heating (blue), particles with direct heating (red), and particles with induction heating (green).

The observed induction heating, 28 °C (Figure 3c), did not reach 80 °C for the rDA release. However, our results confirm biotin released via induction heating. This indicates that localized heating within the particles was achieved while the bulk temperature remained low. Dong and Zink measured core heating within nanoparticles by analyzing the temperature-dependent intensity ratio of emission bands in the upconversion luminescence spectrum of a fluorescent material.^44^ This was achieved using heat induced by superparamagnetic nanocrystals supported on mesoporous silica particles. They found that while high temperatures were induced locally within the nanoparticles, the bulk temperature remained relatively low. Therefore, even when the bulk temperature is low, the core of the magnetic nanoparticles is expected to be sufficiently high to control thermoresponsible reaction.

This work establishes the feasibility of DiMaN as a platform for on-demand, magnetically triggered drug release, and several directions will further strengthen its biomedical potential. Future studies will focus on directly correlating inductive heating efficiency with drug release kinetics, optimizing nanoparticle surface temperatures to improve rDA activation, and systematically assessing in vivo stability and biocompatibility. While biocompatibility testing was not performed here, prior studies have shown that magnetic nanoparticles coated with polymers such as poly­(dimethylacrylamide) exhibit minimal or no acute toxicity.^29,30^ The synthesized diblock copolymer enabled nanoparticle formation and drug conjugation via the DA reaction, while the iron oxide core facilitated magnetothermal heating under an AC magnetic field. Using biotin-PEG_3_-MAL as a model drug provided a convenient proof-of-concept system because the maleimide group allowed efficient conjugation and release, while the biotin moiety served as a quantifiable marker; moreover, biotin is a biologically relevant small molecule (vitamin B7). Moving forward, extending this strategy to maleimide-functionalized therapeutics such as doxorubicin will greatly increase biomedical relevance, allowing in vitro and in vivo studies of therapeutic efficacy and biological response. Although rDA cleavage was observed at ∼80 °C in vitro, which may raise concerns about tissue safety, magnetic induction heating is highly localized at the nanoparticle interface. Supporting this, Attaluri et al. demonstrated that while implanted nanoparticles reached ∼80 °C under an alternating magnetic field, tissue temperatures returned to near-physiological levels within 2 mm of the heated site.^45^ These findings suggest that localized heating sufficient to trigger rDA release can be achieved without widespread tissue damage. With these refinements, DiMaN offers a promising path toward externally controlled, site-specific, and precise drug delivery for applications in oncology and beyond.

## 4 Conclusions

This study demonstrates the successful development of DiMaN, polymer-coated magnetic nanoparticles, as a promising platform for on-demand drug release via magnetic induction heating-triggered retro-Diels–Alder reactions. The system enabled efficient conjugation of maleimide-functionalized drugs, thermally responsive release with retro-Diels–Alder cleavage at elevated temperatures (≥80 °C) while maintaining stability under physiological conditions (37 °C), and confirmation of magnetic induction heating capabilities. Together, these results establish the fundamental chemistry and heating properties of the platform. Looking forward, future work will integrate magnetic field-driven heating with direct drug release, optimize release kinetics, and evaluate biological performance, enabling precise site-specific treatment while minimizing systemic drug exposure and off-target effects. Expanding the approach to include a broader range of conjugated therapeutics, such as chemotherapeutics and biologics, will further demonstrate the system’s versatility for precision medicine applications. With continued refinement, this mNP-based delivery strategy holds strong potential to achieve externally controlled, on-demand drug release, paving the way for highly selective and personalized therapeutic interventions in oncology and beyond.

## Supplementary Material



## Data Availability

The original contributions presented in the study are included in the article/Supporting Information. Further inquiries can be directed at the corresponding author.

## Notes

^1^H NMR for synthesized polymers, polymer synthesis conditions and GPC analysis, GPC chromatogram of p­(DMAm-*co*-FMA)-*b*-pAAc, pH titration results, and iron salt-to-polymer ratios for magnetic nanoparticle synthesis (PDF)
