# Supplementary material for: Magnetic Induction Heating Enables On-Demand Drug Release via Diels–Alder Polymeric Nanocarriers
Source: Biomacromolecules. 2025 Oct 23;26(11):7265–74. doi: 10.1021/acs.biomac.5c00321 (PMC12606646; doi:10.1021/acs.biomac.5c00321)
Supplement: Supplementary file 1 [file bm5c00321_si_001.pdf]

Supporting Information

Magnetic Induction Heating Enables On-Demand Drug Release via Diels-Alder Polymeric Nanocarriers

Nanami Fujisawa,[1,2] Mitsuhiro Ebara,[1,2] and James J. Lai[3,4]*

[1]Research Center for Macromolecules and Biomaterials, National Institute for Materials Science, Tsukuba 305-0044, Japan.

[2]Graduate School of Pure and Applied Sciences, University of Tsukuba, Tsukuba 305-8577, Japan.

[3]Department of Material Science and Engineering, National Taiwan University of Science and Technology, Taipei 10607, Taiwan.

[4]Department of Bioengineering, University of Washington, WA 98195, USA.

*Corresponding author: James J. Lai – Email: jameslai@mail.ntust.edu.tw

(a)

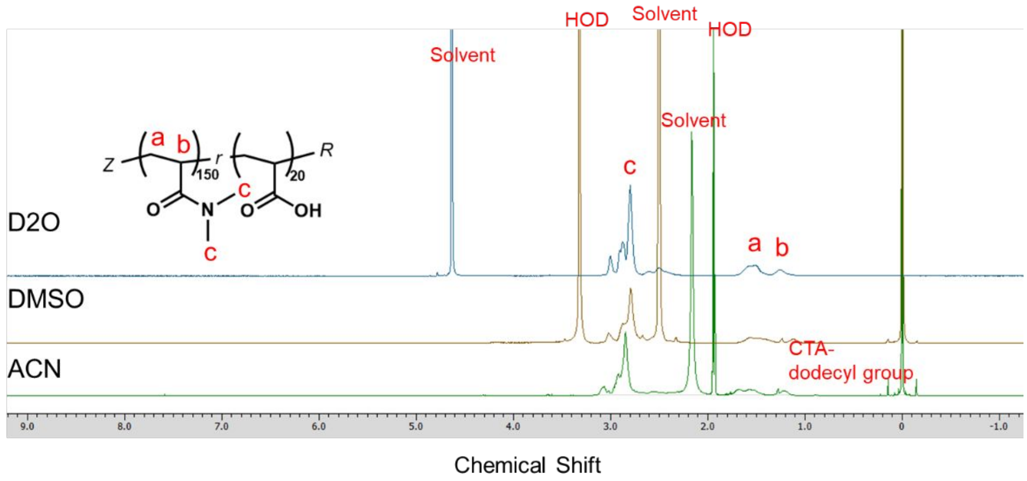
HOD
Solvent
Solvent
HOD
Solvent
c
a
b
D2O
DMSO
ACN
CTA-
dodecyl group
9.0
8.0
7.0
6.0
5.0
4.0
3.0
2.0
1.0
0
-1.0
Chemical Shift

(b)

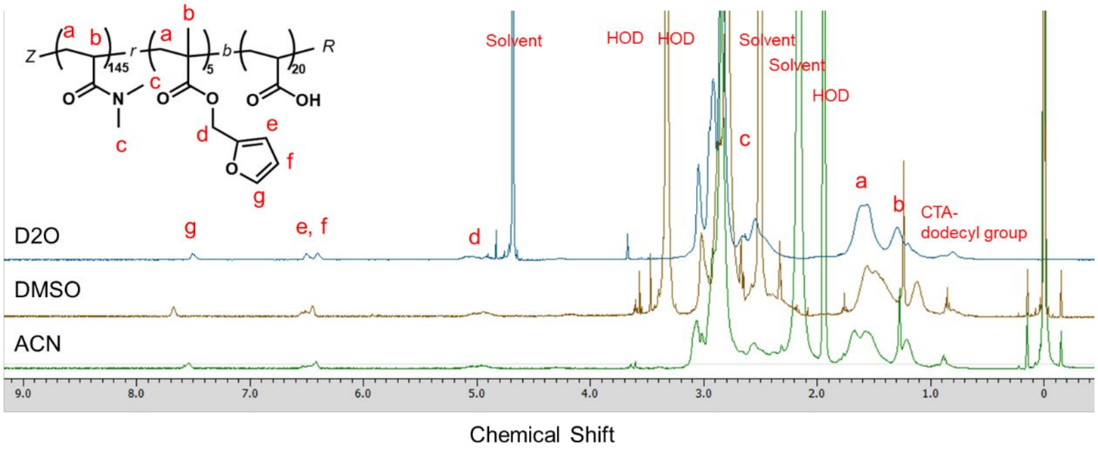
Solvent
HOD
HOD
Solvent
Solvent
HOD
c
a
b
CTA-
dodecyl group
D2O
g
e, f
d
DMSO
ACN
9.0
8.0
7.0
6.0
5.0
4.0
3.0
2.0
1.0
0
Chemical Shift

(c)

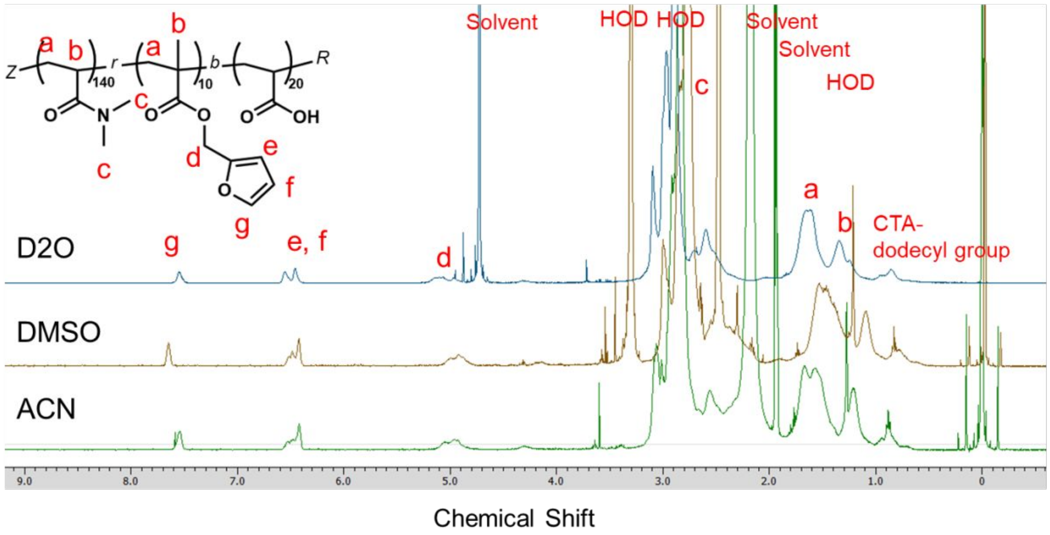
Solvent
HOD
HOD
Solvent
Solvent
HOD
c
a
b
CTA-
dodecyl group
D2O
g
e, f
d
DMSO
ACN
9.0
8.0
7.0
6.0
5.0
4.0
3.0
2.0
1.0
0
Chemical Shift

Figure S1. ^{1}H NMR spectrum at 400 MHz for synthesized polymers. (a) pDMAm-*co*-pFMA, (b) (pDMAm-*co*-pFMA)-*b*-pAAc, and (c) (pDMAm-*co*-FMA)-*b*-pAAc. All polymers were dissolved in a mixture of D_2O, acetonitrile-d_3 (ACN), and DMSO-d_6 at a concentration of 10 mg/mL for measurement.

Table S1. Mn and PDI were measured by GPC in 1.0 mg/mL polymer concentration by RI detector and calculated by polystyrene standard. **Calculated by titration.

Sample name	Solvent	Reaction time	Monomer conc. wt%	DP	AIBN, %(CTA)	Mn	GPC, PDI
($pDMAm_{150}$-*co*-$pFMA_5$)	DMF	4 hr	30	150	0.05	18,600*	1.4
($pDMAm_{145}$-*co*-FMA_5)-*b*-$pAAc_7$	DMF	4 hr	10	20	0.05	19,100**	-

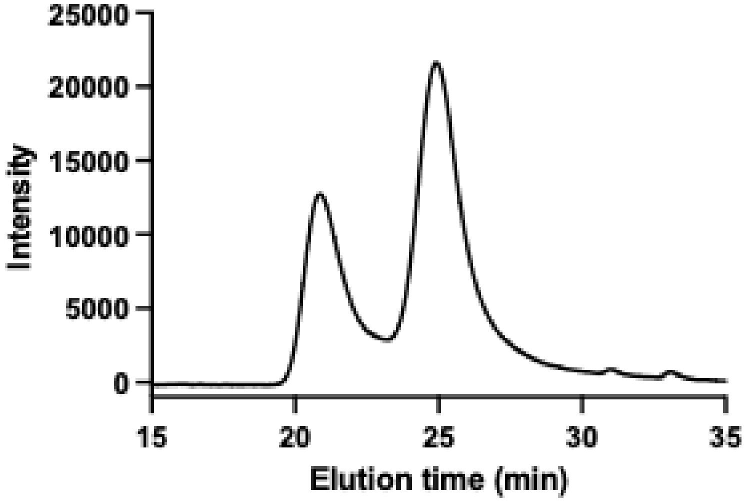

Figure S2. GPC chromatogram of (pDMAm-*co*-FMA)-*b*-pAAc. The mobile phase was 10 mM LiCl in DMF at 40 ℃ with a flow rate of 1.0 mLmin^{-1}, and the elution peaks were detected using an RI detector (RI-501, Shodex, Japan).

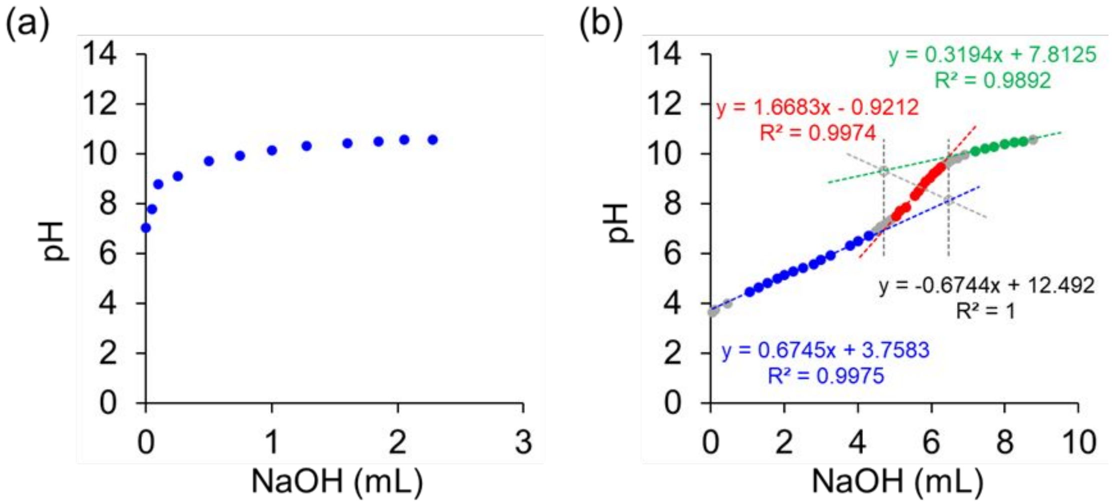

Figure S3. Results of titration of aqueous polymer solution with NaOH solution (0.0104 mol/L) in the sample of pDMAm-*co*-FMA (macro CTA) (a), and (pDMA-*co*-FMA)-*b*-pAAc(b). In (b), the isoelectric point of pKa was 4.4, the amount of carboxylic acid per polymer was calculated from the titration volume at that point to be 7 units of AAc. The experiment was repeated three times.

Table S2. Feed ratio of polymer template magnetic nanoparticle synthesis.

Fe:COOH ratio	Polymer	Polymer	Polymer	Polymer	COOH	Fe2+/Fe3+	Fe2+/Fe3+	Fe2+/Fe3+	MiliQ	Fe3+	FeCl3	FeCl3	Fe2+	FeCl2	FeCl2
	mg/mL	M	μL	mol	mol	mol	μL	M	μL	mol	μL	M	mol	μL	M
20	50	0.002784019	200	5.56804E-07	5.56804E-06	0.000111361	400	0.278401862	400	7.42405E-05	200	0.3712	3.71202E-05	200	0.1856
10	50	0.002784019	200	5.56804E-07	5.56804E-06	5.56804E-05	200	0.278401862	600	3.71202E-05	100	0.3712	1.85601E-05	100	0.1856
5	50	0.002784019	200	5.56804E-07	5.56804E-06	2.78402E-05	100	0.278401862	700	1.85601E-05	50	0.3712	9.28006E-06	50	0.1856
2.5	50	0.002784019	200	5.56804E-07	5.56804E-06	1.39201E-05	50	0.278401862	750	9.28006E-06	25	0.3712	4.64003E-06	25	0.1856
1.25	50	0.002784019	200	5.56804E-07	5.56804E-06	6.96005E-06	25	0.278401862	775	4.64003E-06	12.5	0.3712	2.32002E-06	12.5	0.1856
